# Low dose bitter leaf improves sperm quality disrupted in immunosuppressed Wistar rats: An experimental study

**DOI:** 10.18502/ijrm.v18i3.6720

**Published:** 2020-03-29

**Authors:** Risikat Eniola Kadir, Abdulmumin Ibrahim, Balkis Abimbola Ibrahim, Sadiya Musa Gwadabe, Rukayat Jaji-Sulaimon, Munirat Foyeke Adigun, Adeoye Oyetunji Oyewopo

**Affiliations:** ^1^Department of Anatomy, Faculty of Basic Medical Sciences, University of Ilorin, Ilorin, PMB 1515, Nigeria.; ^2^Department of Human Biology, University of Cape Town, Cape Town, South Africa.

**Keywords:** Bitter leaf, Immunosuppression, Infertility, Prednisolone, Rats.

## Abstract

**Background:**

Synthetic prednisolone (PRED) is a widely used over-the-counter glucocorticoid. Glucocorticoids have inhibitory effects on the immune system and are often used as immunosuppressive agents. Suppressed immunity may impact fertility via the hypothalamic-pituitary-adrenal axis. Bitter leaf (BL) has been shown to improve sperm parameters, but its effects on immunosuppression-associated infertility have not yet been documented.

**Objective:**

To determine the fertility effects of bitter leaf on immunosuppressed Wistar rats.

**Materials and Methods:**

A total of 30 male adult Wistar rats were randomly assigned to 6 groups (n = 5/each). Group A served as a control and were given distilled water in addition to normal feeds, group B received 2 mg/kg PRED for 14 days and served as the standard immunosuppressed group, and groups C-F were immunosuppressed as in B but in addition received 50 mg/kg levamisole, low-dose (250 mg/kg) BL, high-dose (375 mg/kg) BL, and low-dose BL + levamisole, respectively. The CD4 counts, hematological parameters, and sperm parameters were analyzed and compared.

**Results:**

There were significant decreases in sperm motility, progressive motility, morphology, and life/death ratio in the animals given PRED only compared to the controls (p = 0.002, 0.001, 0.001, and 0.01, respectively). These were significantly increased in the treated groups, and animals given levamisole and 250 mg/kg BL showed significantly increased sperm counts compared to the controls (p = 0.04 and p = 0.04, respectively).

**Conclusion:**

Low-dose BL (250 mg/kg) restored the sperm parameters altered by prednisolone administration.

## 1. Introduction 

Synthetic prednisolone (PRED) is a widely used over-the-counter glucocorticoid in Nigeria due to its simple accessibility, low cost, and its fast drug activity which soothes symptoms (1). Glucocorticoids have been shown to be of great clinical importance because of their inhibitory effects on multiple types of immune cells. Hence, they are especially efficacious in managing many of the acute manifestations of inflammatory diseases and autoimmune disorders (2). Since the immunosuppressive effect of glucocorticoids is well established, they are widely used as potent immunosuppressive agents (3). The immunosuppressive action of glucocorticoids is related with numerous possible side effects including infertility and pregnancy through their effect on the hypothalamic-pituitary-adrenal axis (4).

Infertility has been portrayed as an extreme extraordinary issue since it conveys critical psychosocial disturbance, despite the fact that it isn't life threatening (5). A prevalence of 20 - 30% of infertility has been documented in sub-Saharan Africa (6). In spite of the fact that the African sociocultural setting has previously centered around the female, fertility issues are shared by both male and female genders. In Nigeria, male variables contribute 40% to half of the causes of infertility, though with fluctuating incidence and causes from region to region (6). Although the reasons for male infertility are multifactorial, the major cause is hormonal abnormality (7). Hormonal abnormality may be caused by the disturbance or suppression of the hypothalamic-pituitary-adrenal axis, which may arise in part from the uncontrolled or excessive use of glucocorticoids.

Chronic oral ingestion of immunosuppressants has been shown to affect the hypothalamic-pituitary-gonadal axis. This was demonstrated by marked histological changes of testicular structures observed by light microscope (8). Furthermore, degenerative changes of seminiferous tubules and impaired spermatogenesis have been observed in rats exposed to immunosuppressants (8). Similarly, immunosuppressive drugs used for the treatment of medical disorders, such as inflammatory disorders, autoimmune disorders, and transplantation, may have adverse effects on fertility as these have been associated with alterations in hormones that control reproduction (9). The CD4 count has been used as a marker of immunosuppression, and an alteration of hematological parameters, such as RBC count and haematocrit, is observed in patients that are immunosuppressed (10).

Vernonia amygdalina (VA), popularly called bitter leaf (BL) as a result of its bitter taste, is a plant that is broadly devoured in Nigeria. The unpleasant taste of VA is because of its antinutritional constituents such as alkaloids, saponins, glycosides, and tannins (11). It develops prevalently in tropical Africa, particularly in Nigeria, Zimbabwe, and South Africa, and is tamed in parts of West Africa (12). The VA's wood, especially from the root, has been shown to have fertility inducing properties (13).

The fertility effects of BL in laboratory animals have been well documented (14), but data on its effects on infertility associated with immunosuppression is lacking. We hypothesized that BL will improve fertility in rats immunosuppressed with PRED.

## 2. Materials and Methods 

### Collection and extraction of bitter leaf (Vernonia Amygdalina)

Samples of leaves were collected from the botanical garden of the University of Ilorin during October 2016. The leaves were authenticated by an expert botanist in the herbarium of the Department of Plant Biology, University of Ilorin (voucher number: UILH/001/302). The leaves were altogether washed with sterile water to remove stains and afterward air-dried to a consistent weight in the research laboratory. The dried material was pummeled into a dry powder utilizing a mortar and pestle. Two hundred and fifty grams of the powder was extracted with 3.75 L of distilled water as described by Saalu and colleagues (11). The filtrate was concentrated using a rotary evaporator at 50°C until a semisolid residue was obtained. The percentage yield of the extract was then calculated.

### Chemicals (drugs) 

Prednisolone and levamisole (LEV) were purchased at Fiolu Pharmaceuticals Ltd. in Ilorin, a product of Krishat Pharma, Nigeria. PRED was used as the immunosuppressant, while LEV served as the goal standard immunomodulatory agent. PRED and LEV were separately ground into powder forms and separately dissolved in 10 ml distilled water.

### Animal care 

A total of 30 male adult Wistar rats (8 wk old) weighing approximately 150 gr on average were procured from Afolabi farm, Oko Olowo, Kwara State, Nigeria. The rats were bred in the animal house of the College of Health Sciences, University of Ilorin, where they were housed in wooden wire-gauzed and mosquito-netted netted mosquito cages and allowed to acclimatize for 2 weeks under normal atmospheric conditions. The rats were fed with pelletized feed (growers mash) bought from Ogo-Oluwa feeds, Ilorin, Nigeria. Their feeds were put in plates while water was given liberally. The animal care was as stipulated by the National Institutes of Health guide for the care and use of laboratory animals. The animals were weighed at baseline and at the end of the experiment before sacrifice.

### Experimental designs 

The experimental animals were randomized into 6 groups, each consisting of five animals that were randomly selected. Oral administration was completed utilizing a 5 ml syringe and an adaptable feeding tube sufficiently long to get to the stomach through the throat.

The experimental groups were treated as follows:

Group A (control): Received food and distilled water daily. The remaining groups also received the same food and water liberally.

Group B (PRED 2 mg/kg): Received 2 mg/kg b.w prednisolone orally daily for 2 weeks. We used 2 mg/kg b.w PRED for immunosuppression in this study.

Group C (PRED 2 mg/kg + LEV 50 mg/kg): Received oral administration of 2 mg/kg b.w PRED daily for 14 days, followed by daily oral administration of 50 mg/kg b.w of levamisole for 7 days.

Group D (PRED 2 mg/kg + BL 250 mg/kg): Received daily oral administration of 2 mg/kg b.w PRED for 14 days, followed by oral administration of 250 mg/kg b.w of BL for 21 days.

Group E (PRED 2 mg/kg + BL 237 mg/kg): Received daily oral administration of 2 mg/kg b.w PRED for 14 days, followed by oral administrations of 375 mg/kg b.w of BL for 21 days.

Group F (PRED 2 mg/kg + LEV 50 m/kg + BL 250 mg/kg): Received oral administrations of 2 mg/kg b.w prednisolone daily for 14 days, followed by daily oral concurrent administration of 50 mg/kg b.w levamisole and 250 mg/kg b.w of BL for 7 days and then a further administration of 250 kg/mg b.w for 14 days.

### Blood collection 

Blood samples for hematological analysis and CD4 counts were collected retro-orbitally by carefully inserting a heparinized capillary tube into the angle of the eye of rats in groups C to F after 14 days of PRED administration; this was named post-suppression (PS). Post-treatment (PT) blood was collected at the end of the treatment during sacrifice via cardiac puncture. Bloods from groups A and B were collected once, at the time of sacrifice via cardiac puncture.

### Animal sacrifice and tissue processing 

Following the completion of treatment, the rats were weighed and euthanized using 20 mg/kg of ketamine (intraperitoneal), transcardially perfused by injecting 400 ml paraformaldehyde. The testes of each animal were excised; one of each was placed in 5 ml Bouin's solution for fixation and subsequent histopathological examination, and the other was placed in 5 ml physiological saline for further seminal fluid analysis. All animals were sacrificed at day 21, except the animals in group B which were sacrificed earlier on day 14. Histology was performed on paraffin wax-embedded sections in line with the Gray method (15) and stained with hematoxylin and eosin (H&E) as described in Fischer's protocol (16).

### Assessment of hematological parameters and CD4 count

Blood was collected into EDTA heparinized bottles, which were subsequently analyzed for hemoglobin, red blood cells (RBCs), white blood cells (WBCs), platelets, and lymphocyte counts. Analysis was performed using the Sysmex automated hematology analyzer, KX-21N (Sysmex Corporation, Kobe, Japan). An automated Partec Cyflow counter 1, Germany, 2017, was used to determine the CD4 counts.

### Seminal fluid analysis 

Sperm concentration, morphology, and motility were obtained as previously described by Saalu and colleagues (17).

### Histopathological examination and light microscopy 

The paraffin-embedded testes sections were mounted on glass slides, stained with H&E, and cover slipped (15). Sections were captured using an Olympus binocular research microscope connected to a 5.0 MP Amscope Camera (Amscope Inc, USA) and examined for changes in testicular structure.

### Ethical consideration

Ethical approval for this study was obtained from the College of Health Sciences, University of Ilorin Ethical Committee (code: UERC/ASN/2018/1159).

### Statistical analysis

Results were expressed as mean ± SEM. Statistical analysis was performed using statistical GraphPad (USA). Student *t* test and ANOVA were used to compare means, and P-values < 0.05 were taken as significant.

## 3. Results 

### Weight changes of experimental animals

The weights of the animals were checked at the commencement of administration and at the end of treatment just prior to sacrifice. Weight gain was observed in all the groups, though it was only significant in the control, group C, and group D (Table I).

### Effect of prednisolone on CD4 count and hematological parameters

With the exception of group C, there was no significant difference in the PS and PT CD4 count and the RBC count of studied animals. The hemoglobin, WBC count, and neutrophil levels were reduced in all groups studied. Furthermore, the percentage of lymphocytes was higher and neutrophils lower in the control group compared to the treated groups and the PRED only group. The platelet count was increased across all groups, with groups administered BL demonstrating a much higher increase. Detail results in table II.

### Effect of bitter leaf on sperm parameters 

**Figure 1:**
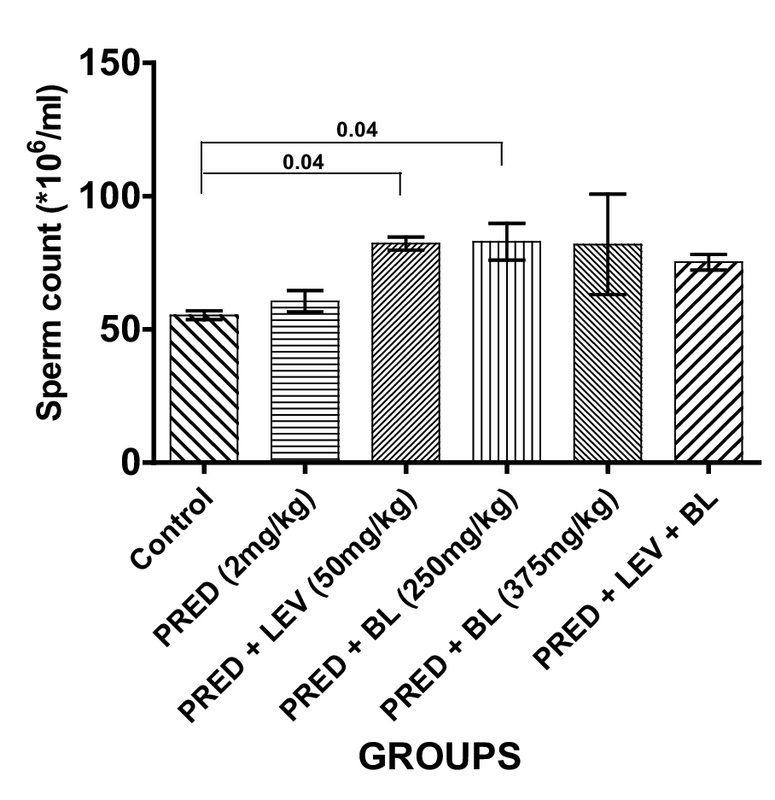
Effect of BL and levamisole on mean sperm count. All data are represented as Mean ± SEM. Analysis of variance (ANOVA) was used to analyze the data at p < 0.05. There was a significant difference between the control and group C (p = 0.04) and between the control and group D (p = 0.04).

**Figure 2:**
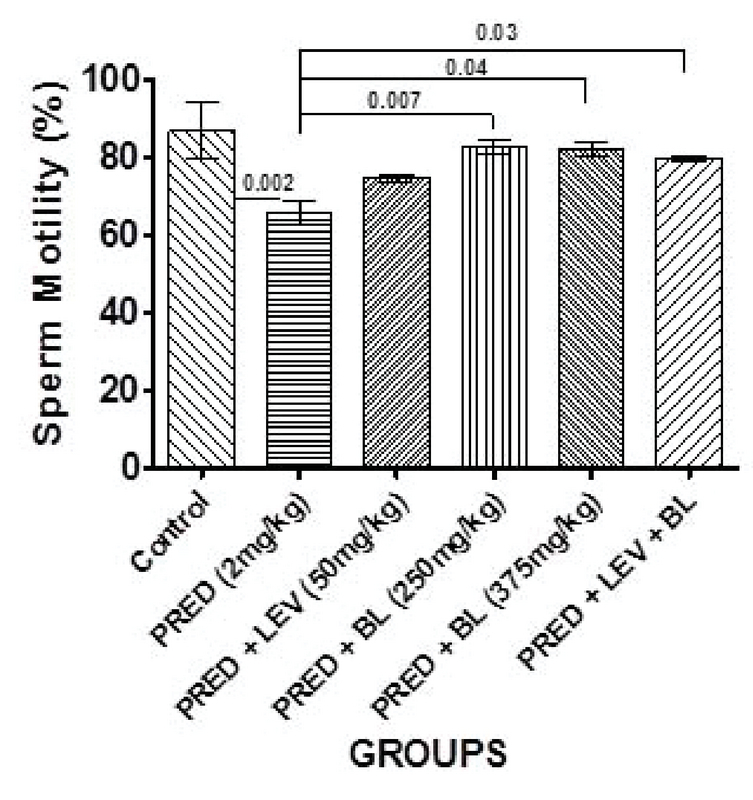
Effect of BL and levamisole on the percentage sperm motility. All data are represented as Mean ± SEM. Analysis of variance (ANOVA) was used to analyze the data at p < 0.05. There was significant differences between the control and group B (p = 0.002), group B and D (p = 0.007), B and E (p = 0.04), and B and F (p = 0.03).

**Figure 3:**
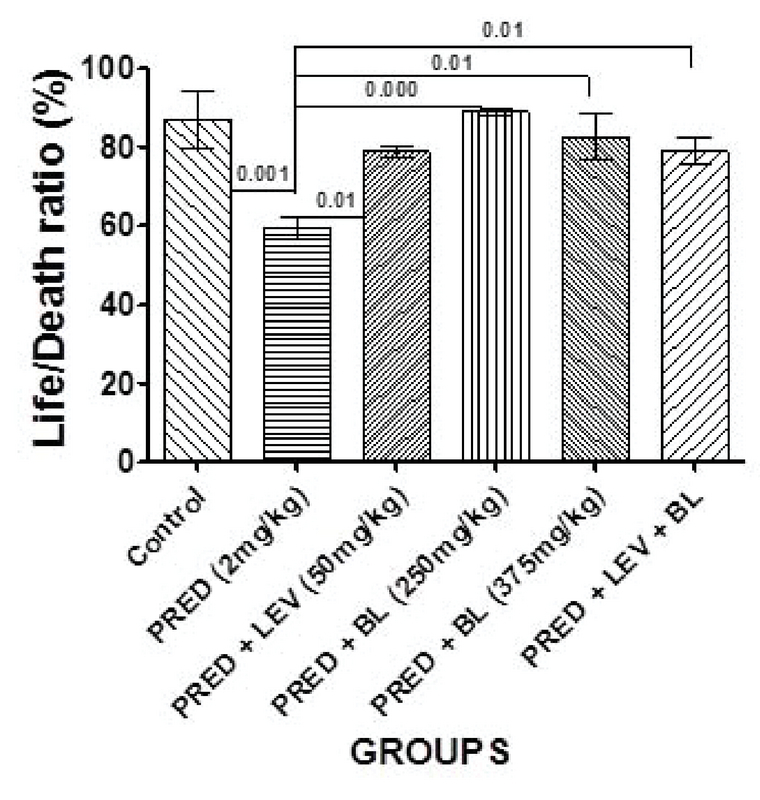
Effect of BL and levamisole on percentage life/death ratio. All data are represented as Mean ± SEM. Analysis of variance (ANOVA) was used to analyze the data at p < 0.05. There were significant differences between the control and group B (p = 0.001) and B and all other treatment groups (C-F) (p = 0.01, p = 0.01, p = 0.000, p = 0.01, and p = 0.01, respectively).

**Figure 4:**
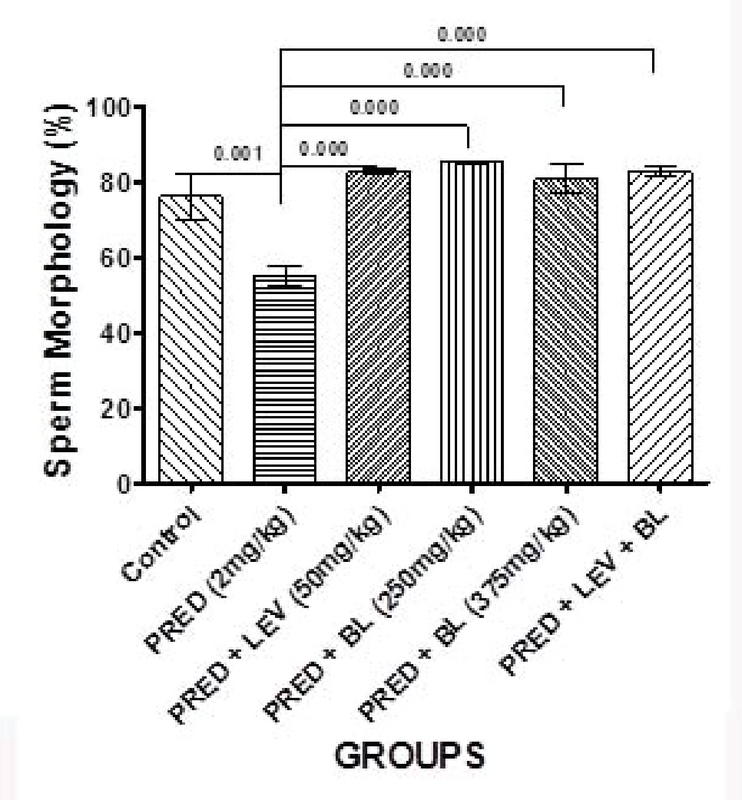
Effect of BL and levamisole on percentage sperm morphology. There were significant differences between the control and PRED (2mg/kg) group (p = 0.001), and PRED (2mg/kg) group and all other treatment groups; PRED + LEV (50mg/kg); PRED + BL (250mg/kg); PRED + BL (375mg/kg) and PRED + LEV + BL at p ≤ 0.001.

**Figure 5:**
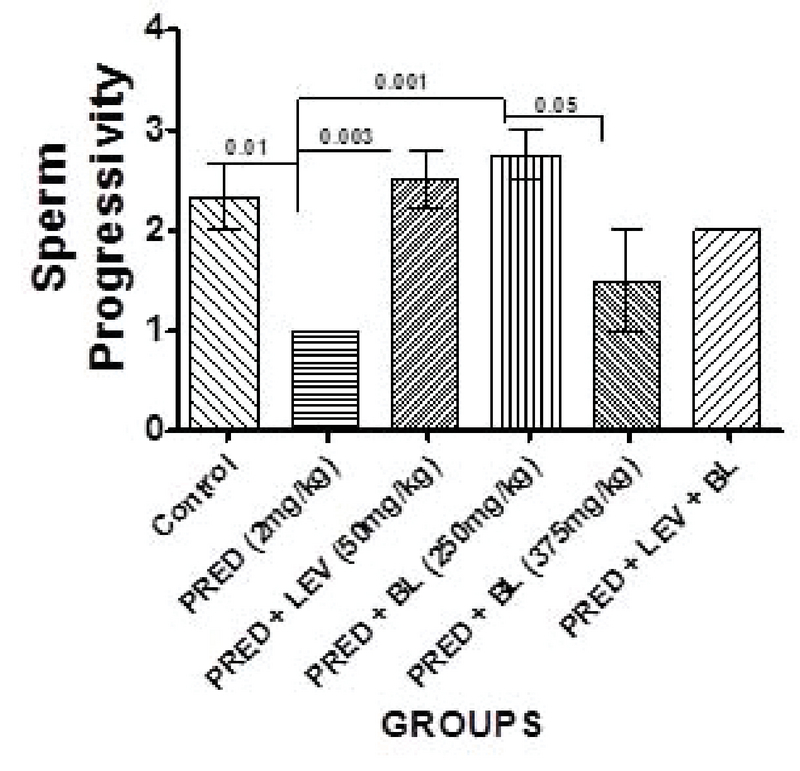
Effect of BL and levamisole on sperm progressivity. There were significant differences between the control and group B (p = 0.01), B and C (p = 0.003), B and D (p = 0.001), and D and E (p = 0.05).

Figure 1-5 show the effect of BL on sperm parameters following the administration of PRED. In Figure 1, Low dose BL improves sperm count significantly when compared to the control. In Figure 2, BL both low and high dose BL increases sperm motility decreased by the administration of PRED. Similarly, Life/death ratio percentages (Figure 3) and sperm morphology (Figure 4) improved in all BL groups compared to PRED only group. High dose BL did not show any significant improvement in sperm progressivity (Figure 5).

### Histopathological examination

**Figure 6:**
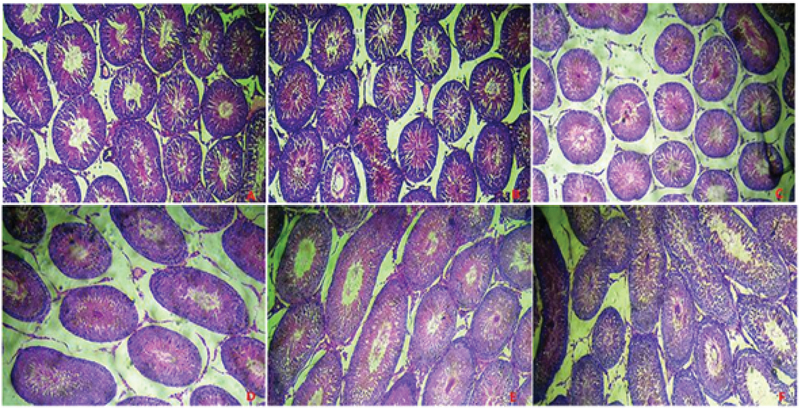
Photomicrographs of transverse sections of the testes of Wistar rats treated with varying doses of prednisolone, levamisole, and BL. (A) Control rats showing a normal arrangement of seminiferous tubules (ST) containing germ cells at different stages of development (spermatogenic series) and prominent interstitial cells. (B) Section of a testis of a prednisolone only treated rat showing less prominent interstitial cells. (C) Section of a testis of a prednisolone and levamisole treated rat showing a widened interstitial space with prominent interstitial cells. (D) Section of a testis of a prednisolone and low-dose BL treated rat showing a widened interstitial space but with prominent interstitial cells like in the control group. (E) Section of a testis of a prednisolone and high-dose BL treated rat showing irregular elongated seminiferous tubules. (F) Section of a testis of a prednisolone plus low-dose BL and levamisole treated rat depicting irregular elongated seminiferous tubules with distorted spermatogenic series. H&E (x100).

Histopathological examination of testicular sections of control rats (A) stained with H&E (x100) showed a normal arrangement of seminiferous tubules (ST) containing germ cells at different stages of development (spermatogenic series) and prominent interstitial cells. The interstitial cells in the group treated with PRED only (B) were less prominent. The groups treated with prednisolone and levamisole only (C) and with prednisolone and low-dose (250 mg/kg w.b) BL only (D) showed a widened interstitial space but with prominent interstitial cells as in the control. Group (E) treated with PRED and high-dose BL (375 mg/kg b.w) and (F) treated with prednisolone, levamisole, and low-dose BL showed irregular arrangement and elongated ST with distorted spermatogenic series (H&E x100) (Figure 6).

**Table 1:** Weight changes of experimental animals


**Groups**	**Initial weight (g)**	**Final weight (g)**	**Weight difference**	**P-value**
**A**	172.7 ± 7.69	203.3 ± 4.41	30.7 ± 8.86	0.03
**B**	162.5 ± 5.56	180.0 ± 4.55	17.5 ± 7.18	0.05
**C**	165.5 ± 1.89	179.3 ± 2.29	13.8 ± 2.97	0.004
**D**	165.5 ± 3.86	195 ± 9.57	29.5 ± 10.3	0.03
**E**	163.6 ± 4.67	179.6 ± 6.79	16 ± 8.24	0.09
**F**	159.2 ± 1.46	174 ± 10.97	14.8 ± 11.1	0.22
Data are represented as Mean ± SEM. Analysis of variance (ANOVA) was used to analyze the data at p < 0.05. A: Control, B: PRED only, C: PRED + levamisole, D: PRED + low-dose BL, E: PRED + high-dose BL, PRED + levamisole + low-dose BL				

**Table 2:** CD4 counts and hematological parameters


	**CD4 (x10${}^{6}$ cells/L)**	**Hemoglobin (g %)**	**RBC (x10${}^{12}$/L)**	**WBC (x10${}^{9}$/L)**	**Platelets (x10${}^{9}$/L)**	**Lymphocytes (%)**	**Neutrophils (%)**
**A**		7.33 ± 1.2	14.30 ± 1.10	8.0 ± 0.61	5.90 ± 1.36	552.0 ± 52.3	77.8 ± 3.72	21.2 ± 3.72
**B**								
	**PS**	7.00 ± 0.71	14.1 ± 0.45	7.90 ± 0.12	7.50 ± 1.79	656.0 ± 84.1	71.3 ± 5.0	27.7 ± 5.0
**C**								
	**PS**	9.75 ± 1.32	15.10 ± 0.55	8.66 ± 0.26	8.85 ± 1.78	585.0 ± 75.9	67.8 ± 7.08	31.20 ± 7.08
	**PT**	4.25 ± 0.48	13.0 ± 0.57	7.80 ± 0.28	6.75 ± 0.58	651.0 ± 92.1	84.2 ± 3.31	14.80 ± 3.31
	**Diff**	5.50 ± 1.40*	1.70 ± 0.79	1.04 ± 0.38*	2.10 ± 1.88	-36.3 ± 119.3	-16.4 ± 7.81	16.4 ± 7.81
**D**								
	**PS**	10.25 ± 1.11	14.20 ± 0.40	8.38 ± 0.23	7.13 ± 1.13	452.0 ± 60.0	62.9 ± 2.04	36.20 ± 2.04
	**PT**	9.75 ± 1.8	12.20 ± 0.49	8.33 ± 0.43	4.83 ± 0.47	636.0 ± 57.1	87.1 ± 1.52	11.90 ± 1.52
	**Diff**	0.50 ± 2.11	2.0 ± 0.63*	0.06 ± 1.24	2.28 ± 1.22	-184 ± 82.8	-24.3 ± 2.55*	24.30 ± 2.55*
**E**								
	**PS**	7.75 ± 1.3	14.10 ± 0.30	7.26 ± 0.57	12.30 ± 1.88	594.0 ± 50.0	65.3 ± 4.45	33.80 ± 4.45
	**PT**	9.0 ± 2.0	11.50 ± 0.70	7.93 ± 0.31	5.70 ± 0.15	801.0 ± 69.0	72.6 ± 7.65	26.50 ± 7.65
	**Diff**	-1.25 ± 2.24	2.58 ± 0.70*	-0.68 ± 0.87	6.63 ± 2.82	-207 ± 86.0	-7.30 ± 8.16	7.3 ± 8.16
**F**								
	**PS**	6.75 ± 0.75	15.10 ± 0.22	8.58 ± 0.22	7.33 ± 1.122	643.0 ± 93.9	62.4 ± 6.14	35.60 ± 6.14
	**PT**	6.25 ± 1.32	12.00 ± 0.30	7.50 ± 0.33	5.53 ± 0.96	770.0 ± 74.1	73.6 ± 7.92	25.40 ± 7.92
	**Diff**	0.5 ± 1.51	3.1 ± 0.62*	1.07 ± 0.40*	1.8 ± 1.56	-128 ± 207.6	-11.2 ± 10.2	11.2 ± 10.02
PS: Post suppression; PT: Post-treatment; Diff: Difference between PS and PT Data are represented as Mean ± SEM. Analysis of variance (ANOVA) was used to analyze the data at p < 0.05. * represents p < 0.05. A: Control, B: PRED only, C: PRED + levamisole, D: PRED + low-dose BL, E: PRED + high-dose BL, PRED + levamisole + low								

## 4. Discussion

To the best of our knowledge, this is the first study to examine the effects of BL on the sperm parameters and histo-architecture of the testes in PRED-immunosuppressed rats. We assayed both the CD4 count and hematological parameters (hemoglobin, RBC, WBC, platelets, lymphocytes, and neutrophils) as a measure of immune health following the administration of PRED. The insignificant differences observed in the hematological parameters and CD4 counts between the control and groups administered with PRED (post-suppression) may not be due to the ineffectiveness of PRED in suppressing immunity in the studied rats. This may point to the fact that the inhibitory effect on the immune system by PRED is, in part, duration dependent as evidenced by the decreased values observed in all post-treatment groups. Neither BL nor levamisole have been shown to have any immunosuppressive properties, and levamisole is a known standard immunomodulatory drug with well-established immunostimulatory properties (18). CD4 T cells are central in the performance of the immune system, as they assist in formation of antibodies together with B cells. CD4 T cells also improve and keep up reactions of CD8 T cells, regulate macrophages, initiate immune responses against many pathogenic microorganisms. CD4 T cells are also significant for immunologic memory, hence a depletion in their numbers result in compromise of the immune system (19).

Therefore, low CD4 counts in immunocompromised individuals imply reduced immune health and have been shown to predispose individuals to opportunistic infections (20) which are preconditions to the disturbance and/or disruption of normal physiology and the general wellness of immunocompromised individuals. The decreased CD4 counts observed in this study show that the immune health of rats is compromised by PRED. Since a low CD4 count has the potential to affect the general function of an organism due to its pivotal role in the control of immunity, we can speculate that the increases and decreases of assayed hematological parameters in our study may be partly in tandem with a decrease in CD4. Low WBC counts and high platelet counts are indicative of a suppressed immune system, usually triggered by immune-targeted disorders such as HIV, autoimmune disorders, lymphoma, liver and spleen diseases, bone marrow disorders, lupus, severe infections, and some medications including high-dose glucocorticoids (21). Thus, our findings of low WBC counts and high platelet counts support this claim. Our findings are also in agreement with those of Ohkaru and colleagues who reported a significant reduction in the number of leukocytes in rats injected with a single dose of dexamethasone, a glucocorticoid drug of the same class as PRED (22). Amar and colleagues also reported a significant increase in the number of platelets in rats that received dexamethasone at the immunosuppressive dose (180 µg/kg/J) after 30 days of treatment (23). Moreover, both PRED and dexamethasone are capable of increasing the platelet count of patients with autoimmune thrombocytopenia (24). However, in contrast to our results, Thanasak and co-workers and Narimane and colleagues reported that the effect of an immunosuppressive dose of dexamethasone in sheep and cows, respectively, was only transient (25, 26). In the current study, we observed an increase in the number of lymphocytes and a decrease in the number of neutrophils; in contrast, Ohkaru and colleagues reported a decreased number of lymphocytes but an increased number of neutrophils. However, Ohkaru and colleagues indicated that the decrease in neutrophil count was due to the neutrophilic mobilization effects of glucocorticoids, whereby apoptosis could be inhibited while promoting the migration of neutrophil into the tissue.

In the present study, PRED was shown to have a significant effect on sperm parameters and may contribute to male factor infertility as evidenced by a significant decrease in sperm motility, sperm morphology, life/death ratio, and sperm progressive motility. Despite these findings, there was no significant difference in the sperm counts of immunosuppressed animals (rats administered 2 mg/kg b.w only) compared to controls. Indeed, rats administered with low-dose BL only and levamisole only had a significantly higher sperm count compared to the controls and the other groups. One study in humans that assessed the effects of an immunosuppressant drug on sperm quality in patients undergoing kidney transplant reported changes in sperm quality with no change in sperm concentration after transplant compared to baseline (27), which is similar to the findings of our study. Furthermore, several other studies also reported similar findings to our study by demonstrating that immunosuppressants have harmful effects, especially on sperm quality (8, 28, 29). However, two (11, 28) of these studies reported significant decrease in sperm count in addition to sperm quality, and this is contrary to our observations in terms of sperm concentration.

Male infertility is related with certain histopathological highlights of the testes, which include loss of sperm and spermatids, a disorganization of germ cell layers, and a nonappearance of germ cell layers (29). The histology of animals given only PRED showed no significant difference compared to the control. This may be due to the short duration between the completion of suppression and sacrifice, which may be too short to show any appreciable histopathological manifestation in the testicular tissue. However, the testicular tissues of animals in groups given high-dose BL and those given concurrent levamisole and low-dose BL showed elongated and seminiferous tubules in disarray with fewer germ cells. Animals given only levamisole, and those given only low-dose BL, did not show pronounced tissue damage apart from a widened interstitial space. However, this may be due to the ongoing repair by BL (low dose) and levamisole.

Our study demonstrates the therapeutic effects of BL on the sperm parameters of the studied animals and highlights that the sperm parameters disrupted by immunosuppressive drugs can be restored using BL because of its special dietary and phytochemical properties that give it various physiological, biochemical, and morphological advantages. Additionally, it is realized that the utilization of vegetables is fundamental for a healthy life because of their antioxidative properties (17).

The ingestion of BL could build up glucose metabolism, prompting the creation of pyruvate which is known to be the favoured substrate fundamental for the movement and survival of sperm cells (30). As antioxidants, the flavonoids and nutrients in BL concentrate could keep up sperm morphology, sperm survival as well as its functions. In this study, animals treated with BL showed improvement in sperm motility, normal morphology and number of live sperm cells. Findings in this study conforms with studies by Oyeyemi and coleagues, suggesting increases in sperm quality with administration of BL (14), who reported significant improvement in all the sperm parameters of rats treated with BL. However, in Oyeyemi, in our study, high-dose BL significantly decreased sperm progressivity; this is in line with the findings of Igile co-workers (31). The results of our study also showed that animals treated with a standard immunomodulatory agent (50 mg/kg levamisole only) and their counterparts treated with 250 mg/kg BL show an almost equal effect on sperm parameters and histological appearance, as well as significant steady weight gain (similar to that of the control animals). However, concurrent combination of the two (250 mg/kg BL and 50 mg/kg levamisole) caused a decrease in the sperm motility and damaged testicular tissue. Our results on rats treated with 50 mg/kg levamisole only oppose the work by Bozkurt and coworkers that reported that levamisole caused a significant decrease in semen volume, sperm motility, concentration, and total sperm number at all times (32). However, it is important to note that their study was on rams and with a dose of 7.5 mg/kg.

## 5. Conclusion

The results obtained from our study show that PRED, at an immunosuppressive dose, significantly affects normal sperm parameters. Despite this finding, we could not make a generalizable conclusion on its effect on the cytoarchitecture of testicular tissues. However, although the affected sperm parameters were better restored by administering 250 mg/kg BL, a higher dose of the BL could be deleterious to the sperm progressivity. In addition, similar studies considering sex hormone profiling and inflammatory immune markers, such as IL-1 and IL-6, would give a better understanding of the effects and/or role of the immune system on the hypothalamic-pituitary-gonadal axis. This would be an interesting addition to the current literature on the effects of immunosuppression on male fertility.

##  Conflict of Interest 

The authors have no financial relationships to disclose or conflicts of interest to resolve.
